# Analysis of Enterovirus 68 Strains from the 2014 North American Outbreak Reveals a New Clade, Indicating Viral Evolution

**DOI:** 10.1371/journal.pone.0144208

**Published:** 2015-12-02

**Authors:** Juan Du, Baisong Zheng, Wenwen Zheng, Peng Li, Jian Kang, Jingwei Hou, Richard Markham, Ke Zhao, Xiao-Fang Yu

**Affiliations:** 1 Institute of Virology and AIDS Research, First Hospital of Jilin University, Changchun, Jilin, China; 2 Department of Molecular Microbiology and Immunology, Johns Hopkins Bloomberg School of Public Health, Baltimore, Maryland, United States of America; Shanghai Medical College, Fudan University, CHINA

## Abstract

Enterovirus 68 (EVD68) causes respiratory illness, mostly in children. Despite a reported low-level of transmission, the occurrence of several recent outbreaks worldwide including the 2014 outbreak in North America has raised concerns regarding the pathogenesis and evolution of EVD68. To elucidate the phylogenetic features of EVD68 and possible causes for the 2014 outbreak, 216 EVD68 strain sequences were retrieved from Genbank, including 22 from the 2014 outbreak. Several geographic and genotypic origins were established for these 22 strains, 19 of which were classified as Clade B. Of these 19 strains, 17 exhibited subsequent clustering and variation in protein residues involved in host-receptor interaction and/or viral antigenicity. Approximately 18 inter-clade variations were detected in VP1, which led to the identification of a new Clade D in EVD68 strains. The classification of this new clade was also verified by the re-construction of a Neighbor-Joining tree during the phylogenetic analysis. In addition, our results indicate that members of Clade B containing highly specific alterations in VP1 protein residues were the foremost contributors to the 2014 outbreak in the US. Altered host-receptor interaction and/or host immune recognition may explain the evolution of EVD68 as well as the global emergence and ongoing adaptation of this virus.

## Introduction

Human Enterovirus 68 is a member of the genus *Enterovirus* that belongs to the family *Picornaviridae*. Human enteroviruses can be classified into four different groups (HEV-A to HEV-D) based on serological and phylogenetic analyses [1]. Enterovirus 68 has been classified as a member of group D and thus designated as EVD68. In contrast to other common enteroviruses such as Enterovirus 71 (EVA71) and Coxsackievirus A16 (CV-A16), both of which are categorized as HEV-A and are responsible for the widely spread hand, foot, and mouth disease (HFMD) [1–9], EVD68 causes respiratory illness with mild (sore throat, cough, and fever) to serious (including dyspnea or even respiratory failure) symptoms [10–19].

Similar to other enteroviruses, EVD68 is a non-enveloped virus containing a ~7.4-kb RNA genome coding for one polyprotein. Subsequent to translation by the host machinery, this polyprotein is self-digested by viral proteins 2A and 3C yielding four structural proteins (VP1, VP2, VP3, and VP4) and seven non-structural proteins (2A, 2B, 2C, 3A, 3B, 3C, and an RNA-dependent RNA polymerase). Although all structural and non-structural proteins are important for the replication of EVD68, viral proteins VP1-4 participate directly in the assembly of new EVD68 virions as well as in host-receptor recognition during further rounds of infection [20].

EVD68 infections were first detected in 1962 [21] and were considered rare until the recent worldwide occurrences of clusters and outbreaks [15, 22–31]. These outbreaks most likely indicate that EVD68 has continued to evolve over the past decades, resulting in increased viral infectivity, replication potency, and/or host immune evasion thus facilitating viral transmission. Previous analysis indicated that the most recent common ancestor (TMRCA) of EVD68 existed around 1960 [26], indicating that EVD68 is a relatively new enterovirus, compared to Enterovirus 71 (TMRCA arose in 1931) [4] or Coxsackievirus A16 (first isolated in 1951) [32], and that EVD68 continues to evolve and adapt to its host.

The recent EVD68 outbreaks indicate that EVD68 is well adjusted to transmission across hosts; the most recent outbreak occurred in North America in 2014 (the 2014 outbreak); over a thousand children in the US and another hundred in Canada were infected, with a minimum death toll of 14 [33]. Although similar outbreaks have been reported, as well as the involvement of EVD68 viral sequences [34, 35], there were no obvious cause(s) for the occurrence of the 2014 outbreak. Thus, phylogenetic analysis of EVD68 strains from the 2014 outbreak could provide up-to-date information regarding the evolution status of the virus.

In this study, we analyzed the VP1 region of 216 EVD68 strains by phylogenetic methods, including 22 from the 2014 outbreak. We determined that strains of Clade B EVD68 that were not detected in US in the past contributed most to the recent outbreak in 2014. Further analyses of VP1 variation indicated that altered viral antigenicity was among the potential causes of the 2014 outbreak. Meanwhile, we also detected a new clade, namely Clade D, based on several criteria for EVD68 clade classification. Our study therefore provides explanations for the occurrence of the 2014 outbreak and reveals the most recent evolutionary status of EVD68, which are essential for future control and prevention of EVD68 transmission.

## Materials and Methods

### Sequences

Initially, we intended to use the full-length VP1 region of EVD68 (nucleotides 2389–3315, corresponding to the EVD68 Fermon sequence [accession number, AY426531]) for this study, since it best correlates with the antigenicity of enteroviruses and has been widely used for enteroviral subtyping. However, in order to include additional EVD68 sequences without losing the number of sites required for the subsequent phylogenetic analyses, we decided to use a shorter region (2476–3129). Based on these criteria, 216 EVD68 sequences, including 22 from strains isolated during the 2014 outbreak, were retrieved from GenBank (S1 Table). However, full-length VP1 region of fewer (117 strains) EVD68 sequences were tested during some of the analyses, which is clearly indicated.

### Bayesian analysis

Bayesian evolutionary analysis was performed using procedures similar to previously described methods [26]. Briefly, the retrieved EVD68 sequences were first aligned with MEGA5 (version 5.2.2) [36]. The evolutionary relationships of the viral sequences, along with TMRCA, were then inferred using the Bayesian Markov chain Monte Carlo (MCMC) method implemented with the BEAST package (version 1.8.1) [37]. The uncorrelated log-normal relaxed clock was used with an Extended Bayesian Skyline Plot and both HKY and SRD06 models for nucleotide substitution. The MCMC chains represent 200-million-iterations and the parameters were subsampled every 10,000 iterations.

The collected data were first evaluated with Tracer (version 1.6) to calculate the effective sample size (ESS); an ESS >300 was required for each of the parameters in order to confirm sampling efficiency. Next, TreeAnnotator (version 1.8.1) was used to generate a Bayesian evolutionary tree, removing 10% burn-in and summarizing the maximum clade credibility trees. Finally, the tree was visualized and modified with FigTree (version 1.4.2).

### Phylogenetic analyses

To confirm the clade classification, a Neighbor-Joining tree was re-constructed with full-length VP1 sequences. In total, 117 EVD68 VP1 sequences were used and first aligned with MEGA5 [36]. The Neighbor-Joining tree was then re-constructed by using the Kimura-2-parameter model. Additional parameters were included during the re-construction to correlate with those for the Bayesian evolutionary analysis above: Gamma Distributed rates were applied with a parameter of 4, and only the 1st and 2nd positions of each codon was used. The re-constructed tree was then subjected to a phylogeny test based on the bootstrap method for 1,000 replicates.

To reveal possible evolutionary selection of each codon of EVD68 VP1, strains from each clade were aligned and the values of dN-dS for each codon were calculated with MEGA5 [36].

### Protein remodeling

The structure of EVD68 VP1 was based on the published structure of the EVD68 virion (PDB: 4WM8) [20]. Remodeling and color manipulation were performed using PyMOL software (http://www.pymol.org, version 1.7.6.0).

In addition, the structure of Echovirus 7 (PDB, 2X5I) [38] was used as the template to reconstruct the structure of EVD68 with additional intact loop regions. The structure was proposed with MODELLER software (version 9.14) [39] using CA/AFP/v14T04344 (accession number, KM892502) as the subject. Remodeling, structural alignment, and color manipulation were performed using PyMOL.

## Results

**Fig 1:**
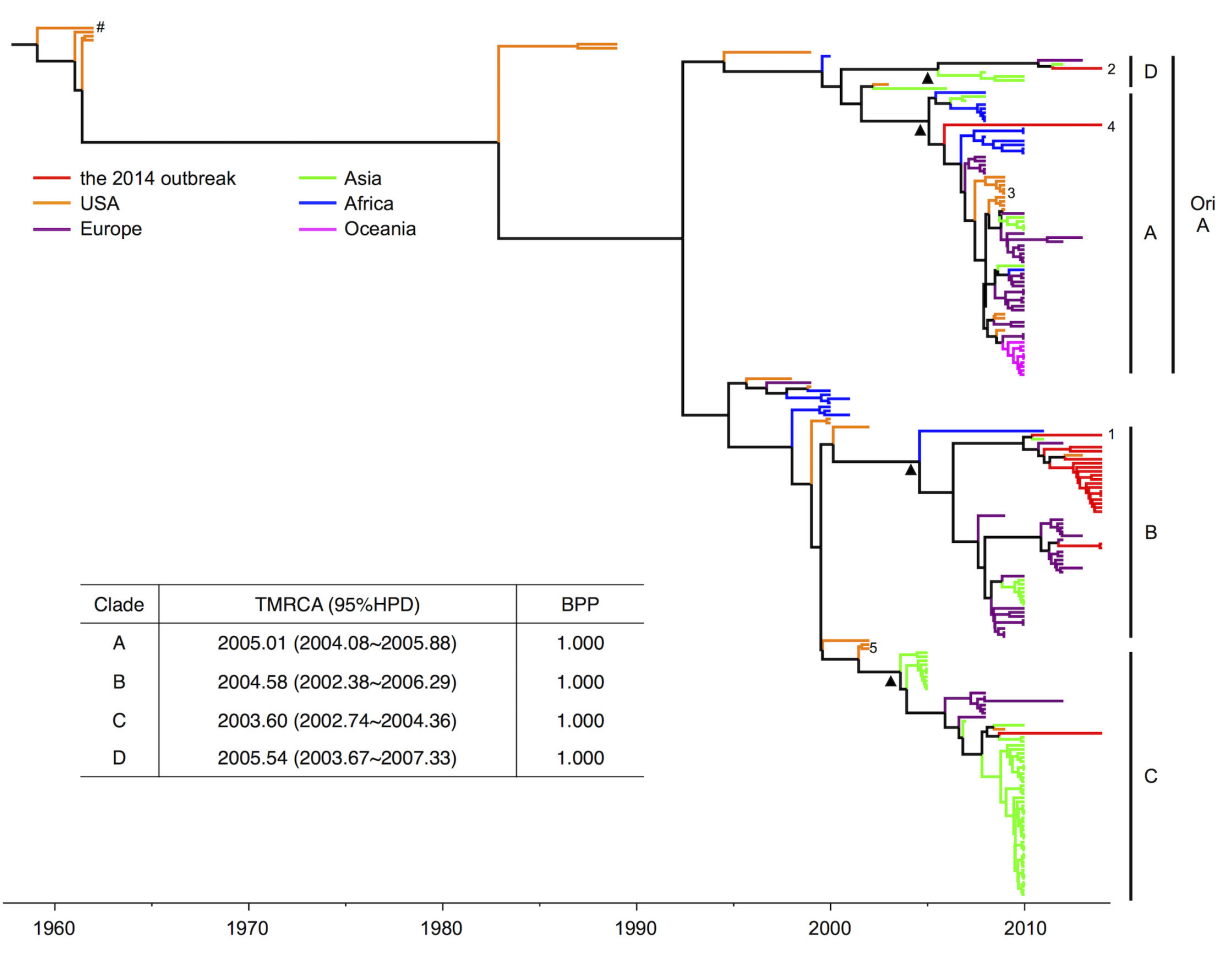
Bayesian MCMC analysis revealed Clade B members as the key contributors to the 2014 outbreak and demonstrated the emergence of a new Clade D from the original Clade. **A.** Reported EVD68 sequences retrieved from GenBank are labeled in different colors in this tree based on sample origin, with the 2014 outbreak strains in red. The Bayesian phylogenetic tree was reconstructed with the partial VP1 coding region (2,435–3,128) according to a previously described method [26]. The original Clade A (ori A) classification is shown in addition to the four clades classified in this study. The scale bar below the tree indicates the time of strain sampling. Triangle markers indicate nodes for new clade classification. Specific strain labeling: Hashtag, EVD68 Fermon strain; 1, CA/AFP/11-1767; 2, US/KY/14-18953; 3, NYC strains; 4, CA/RESP/10-786; 5, MD02-1 and MD02-2. The year of the most recent common ancestor (TMRCA) and the Bayesian posterior probability (BPP) for each clade are shown in the table.

To gain a general understanding of the evolutionary status and viral relationships, a Bayesian phylogenetic tree was reconstructed using BEAST (version 1.8.1) [37] based on 216 published EVD68 VP1 sequences, including 22 sequences from the 2014 outbreak (Fig 1). Addition of the 2014 outbreak strains did not significantly alter the estimated year of TMRCA (1959.09, with a 95% highest probability density [HPD] of 1950.39–1961.34), compared to previously published studies [26]. Interestingly, the 2014 outbreak strains were located in several different clusters (Fig 1), with two strains being classified as the original Clade A, one as Clade C, and the remaining 19 as Clade B. The clustering of the 2014 strains with strains isolated outside of the US also suggested that the strains that caused the 2014 outbreak had multiple origins.

**Fig 2:**
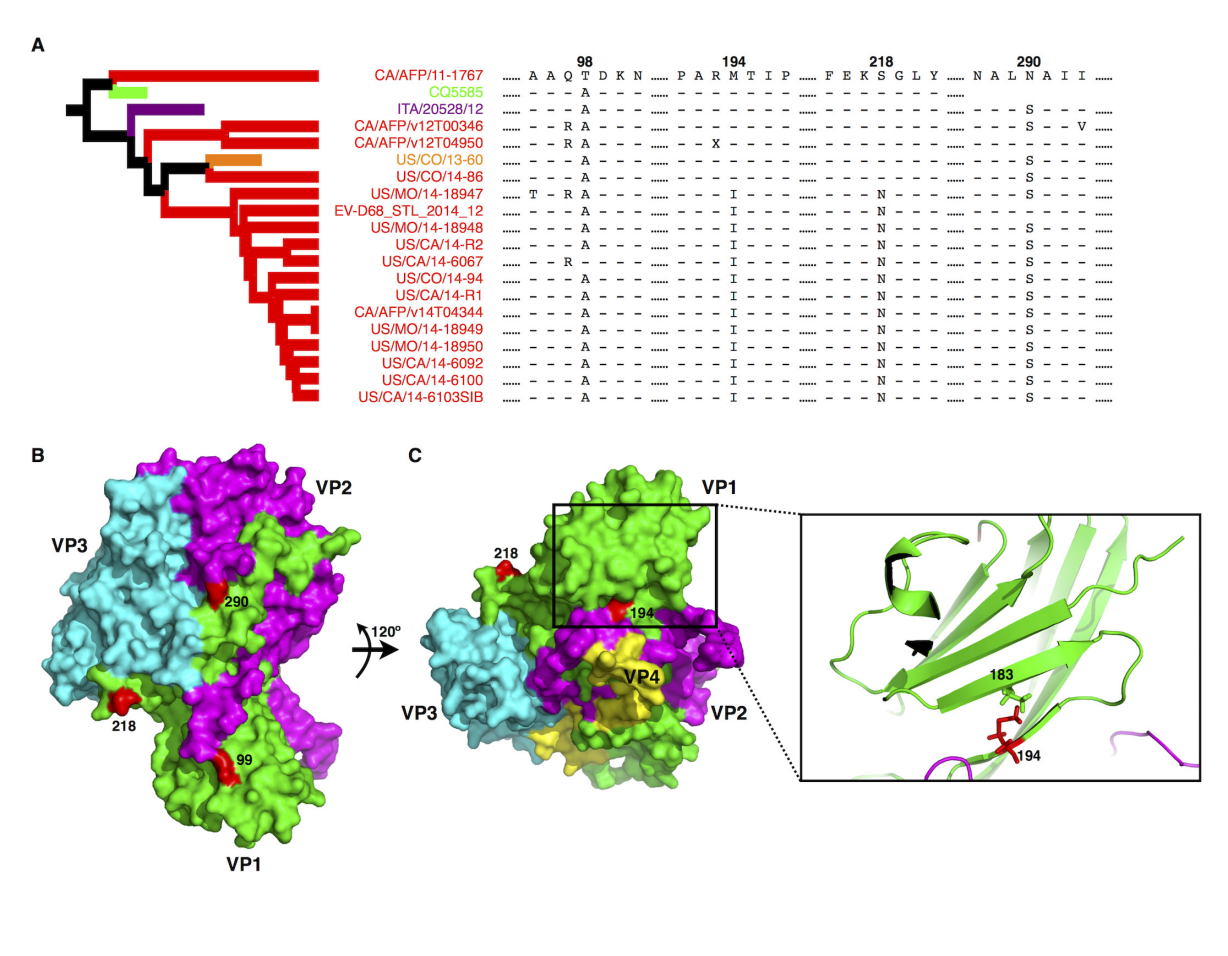
Specific residue variations detected in the EVD68 VP1 protein from the 2014 outbreak strains. **(A)** VP1 protein alignment based on the Clade B subcluster. The alignment was performed with MEGA5 [36]. Hyphens indicate residues identical to those in CA/AFP/11-1767; “X” indicates unidentified amino acid due to unidentified nucleotides in the submitted CA/AFP/v12T04950 sequence. Numbers indicate protein positions. Sequence CQ5585 is missing the fragment surrounding position 290. The subtree for the subclustered Clade B strains is extracted from the tree in Fig 1. (**B)** Structures of viral proteins VP1 (green), VP2 (purple), and VP3 (cyan) in the surface model. The remodeling was based on the published EVD68 structure (PDB: 4WM8) [20]. Positions 218 and 290, located on the surface of VP1, are labeled in red. Position 99 is shown in red instead of 98 since the latter is missing in the published EVD68 structure. (**C)** Position 194, is located beneath the viral surface and is essential for β-sheet formation, which may be important for supporting EVD68 virion “canyon” formation. Left panel: the surface model of VP1 rotated 120° from the model shown in Fig 2B; right panel: a cartoon model of the β-sheet in which residue 194 interacts with residue 183.

Of the 19 Clade B strains that predominately contributed (86.36%) to the last known EVD68 outbreak in North America in 2014, 17 belonged to a subclade that separated from other strains around the year 2007 (Fig 1). The clustering of this subclade was confirmed with a Bayesian Posterior Probability (BPP) value of 1. Moreover, among these 17 strains, CA/AFP/11-1767 (strain number 1 in Fig 1) was a relatively early strain, indicating that the remaining 16 might be evolutionary progeny. A more detailed examination of the VP1 protein sequences indicated that CA/AFP/11-1767 contains two residues at positions 98 and 290 that differ from those found in most B1 strains (Fig 2A, position 99 is indicated instead of 98 since the latter was missing from the published structure). Interestingly, based on published structural information for EVD68 [20], position 290 occupies a surface position that is mostly likely linked with the antigenicity of VP1 (Fig 2B). Position 98 is located on a loop region surrounding the icosahedral five-fold axes (or the “canyon” region) responsible for host-receptor interaction (Fig 2B). The changes to both residues altered the length of the side chain; the change at position 98 also changed the polarity of the side chain (Fig 2A).

Similar residue substitutions occurred at positions 194 and 218 in most of the remaining B1 strains (12 of 16; 75.00%; Fig 2A). In contrast to positions 98 and 290 that are located on the central surface region, position 218 is located on the side of VP1 and is possibly involved in VP1-VP1 interactions and hence in “canyon” formation (Fig 2B). Position 194 is not located on the surface of EVD68 but rather faces inward (Fig 2C). Despite the moderate decrease in the length of the side chain, the alteration from methionine to isoleucine at position 194 is highly conserved in these 12 B1 strains. Furthermore, the cartoon model indicated that although position 194 was very close to VP2 (Fig 2C), it was not involved in VP1-VP2 interactions. Rather, it participated in the formation of a β-sheet connected to loops that contributed to “canyon” formation (Fig 2C). Importantly, the alterations at positions 194 and 218 seem to co-exist in addition to the changes at positions 98 and 290 (Fig 2A), suggesting a possible synergistic effect on the binding of VP1 to the cellular receptor. Therefore, the 2014 outbreak was very likely due to modification of the receptor interaction affecting local cell tropism; the inability of previously established anti-EVD68 antibodies to detect the altered residue at position 290 might have contributed to the occurrence of the outbreak.

Although other EVD68 clades contributed slightly to the 2014 outbreak, one of the three non-Clade B strains, US/KY/14-18953 (strain number 2 in Fig 1), piqued our interest. This strain was first determined as belonging to Clade A based on a previous classification [26], however it was evolutionarily distant from other Clade A strains isolated in the US, such as the NYC strains from 2009 (orange color in Clade A, Fig 1) and CA/RESP/10-786 from 2014 (strain number 4 in Fig 1). Moreover, unlike previous reconstructions of EVD68 phylogenetic trees [26, 30], we detected an additional cluster with a BPP value of 1 that included strain US/KY/14-18953 (Fig 1). Most of the remaining Clade A sequences formed another cluster (Fig 1) originating in 2001; this new clade also had a BPP of 1.

**Fig 3:**
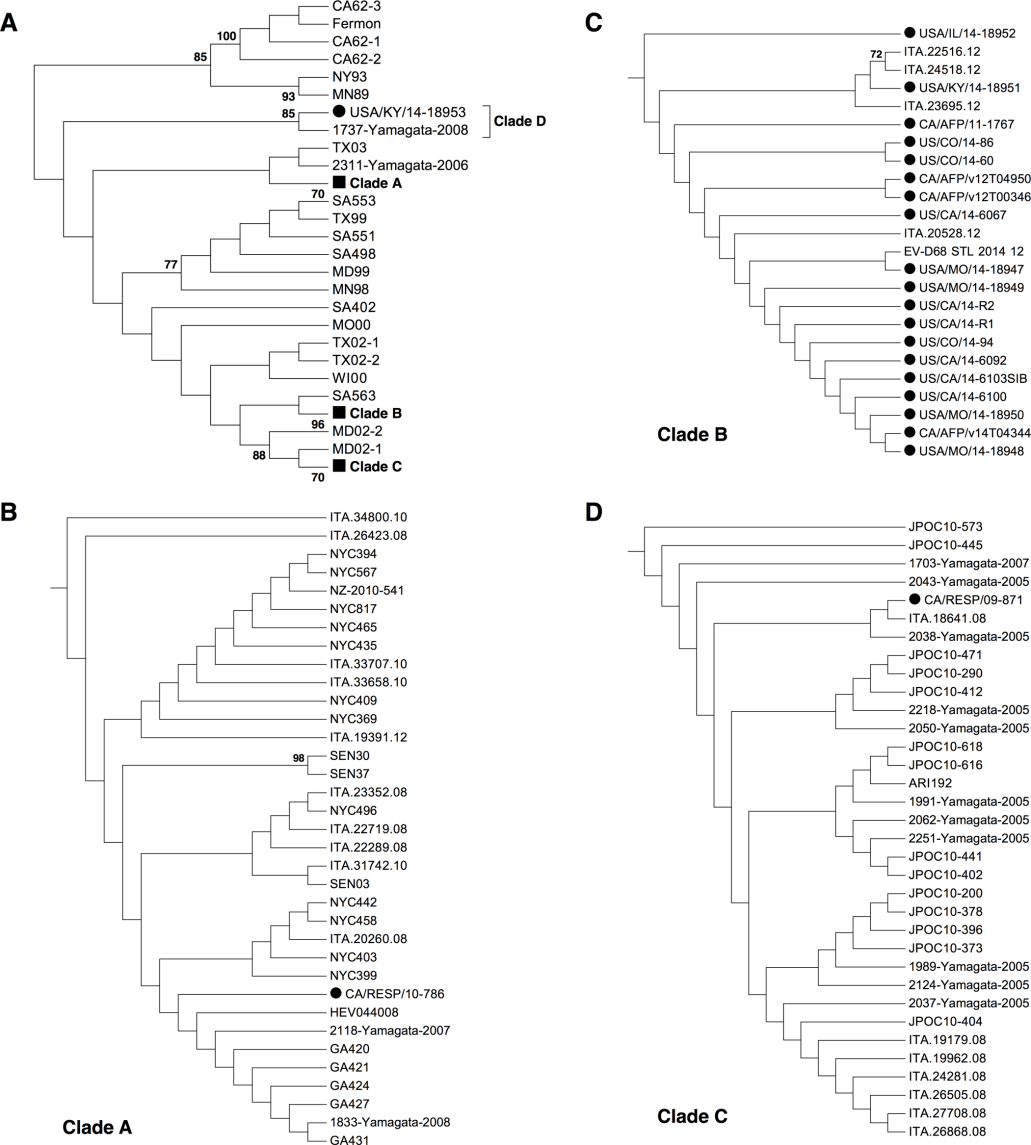
Phylogenetic analysis of full-length VP1 region of EVD68 strains. 117 full-length EVD68 VP1 sequences were used for the re-construction of the Neighbor-Joining tree (**A**). Similar clade formation was detected compared to that in Fig 1, and each clade was shown as a subtree (**B, C, and D,** for Clade A, B, and C respectively). The tree was re-constructed with the help of MEGA5 [36]. Only bootstrap values >70 were shown. For a clearer presentation of the relationship between strains, only the topology was shown. Circle symbols indicate EVD68 strain from the 2014 outbreak, while square symbols indicate compressed EVD68 clades.

Full-length VP1 is used most frequently to determined the subtype for other enteroviruses such as EVA71 and CA16. To avoid any bias introduced to the Bayesian analysis above based on only a partial VP1 sequence, a different approach based on full-length VP1 was necessary to confirm the separation from Clade A. Out of 226 strains used in this study, 117 of them contained full-length VP1 and these full-length VP1 sequenes were then subjected to a Neighbor-Joining (NJ) tree re-construction with MEGA5 [36] (Fig 3). Interestingly, a more significant separation from Clade A was observed, and the cluster containing USA/KY/14-18953 was phylogenetically distant from the remaining Clade A strains (Fig 3A and 3B). On the other hand, the NJ tree presented clustering of Clade A, B, and C, similar to that detected in the Bayesian analysis (Fig 3B, 3C and 3D), confirming the validity of the NJ tree re-construction. In addition, an amino acid comparison of the full-length VP1 regions showed that the difference between the two Clade A clusters were similar to those between the other clades (Table 1), indicating that US/KY/14-18953 belongs to a new clade (Clade D, Fig 1). Clade D is distinct from Clade A and emerged during recent instances of EVD68 transmission.

**Table 1:** Amino acid variation in the VP1 residues of different EV68 clades.

Clade	A	B	C	D
**A**	-	5.56%	4.58%	4.58%
**B**	17 (12–24)	-	4.58%	5.88%
**C**	14 (15–20)	14 (10–21)	-	5.23%
**D**	14 (10–23)	18 (16–21)	16 (14–20)	-

The comparison was performed with the full-length EV68 VP1 region (306 amino acids) from 117 EVD68 sequences. The clade classification is shown in Fig 1. Values in the lower-left indicate the average number of different amino acids between the clades, with the range in parentheses. Values in the upper-right indicate the relative percentage of the VP1 region that differs among the clades. Sequence alignment and calculation were performed using MEGA5 [36].

**Fig 4:**
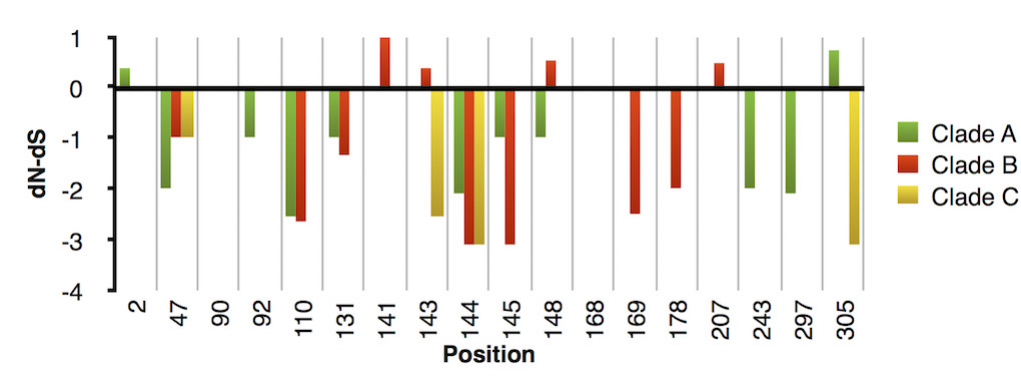
Evolutionary selection on the 18 clade-specific positions. After sequence alignment, the dN-dS values for each clade were calculated for these clade-specific positions with the help of MEGA5 [36]. Values >0 indicate positive selection, while values <0 indicate purifying selection. Each codon with dN-dS = 0 in this study shared conserved nucleotides and thus was considered as purifyingly selected. Clade D was excluded from this analysis because only two strains contained full-length VP1.

To further confirm the accuracy of our new clade classification, the primary protein sequences of the VP1 region were compared in detail; these sequences have been used in the phylogenetic classification of EVD68, which correlated with the serological classification. In total, 18 positions with amino acids that differed among the clades were detected (Table 2). A purifying selection was observed for each of these 18 positions within at least each of two clades, where a dN-dS value ≤0 was observed (Fig 4). Therefore, the pattern for both intra-clade conservation and inter-clade variation in Table 2 validated the novel clade classification.

**Table 2:** Specific amino acids for EV68 clade classification.

Clade	Position																	
	2	47	90	92	110	131	141	143	144	145	148	168	169	178	207	243	297	305
A	D	S	N	A	K	V	-	S	N	S	T	E	K	A	V	I	D	N
D	E	S	N	T	K	I	-	N	N	S	M	E	K	G	V	I	E	D
B	D	T	D	T	R	I	G	S	N	N	V	E	K	G	V	V	D	N
C	D	T	N	T	R	I	G	S	S	N	M	K	Q	G	I	I	D	N

Amino acids differing among the clades were compared based on an alignment of the full-length VP1 sequence performed using MEGA5 [36] with manual adjustment. Clade classification is shown in Fig 1. The numbers indicate residue position corresponding to the VP1 protein of the Fermon strain.

**Fig 5:**
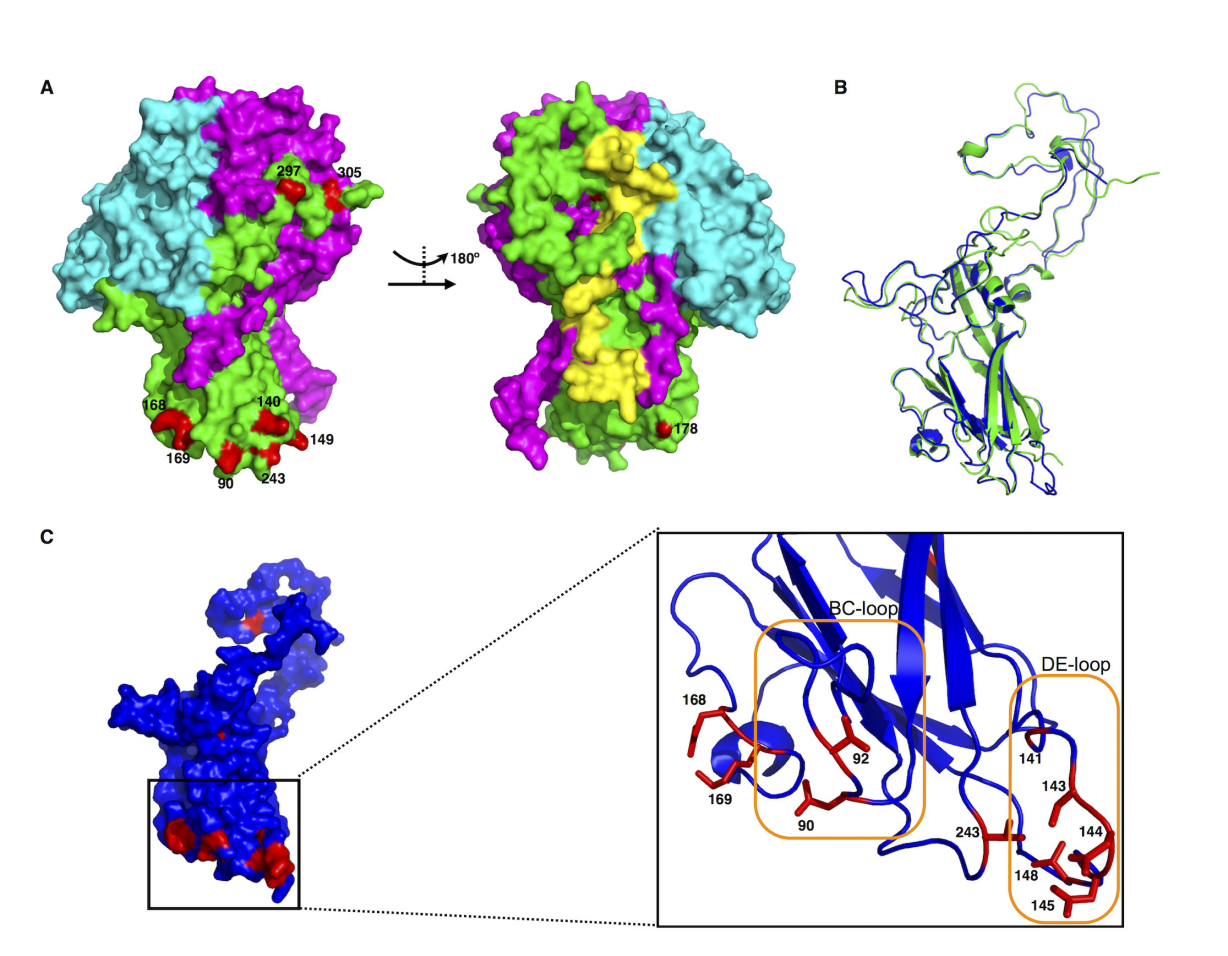
Host-receptor binding and/or viral antigenicity correlate with EVD68 clade classification. **(A).** Surface models of EVD68 VP1 (green), VP2 (purple), and VP3 (cyan), with clade-specific residues labeled in red. The remodeling was based on the published EVD68 structure (PDB: 4WM8) [20]. Positions 140 and 149 are labeled in red to indicate the approximate positions of residues 141, 143, 144, 145, and 148 that are missing in the published structure. **(B).** Structural alignment of VP1 from the published EVD68 structure (green) or the proposed structure (blue) based on the viral structure of Echovirus 7 (PDB: 2X5I) [38]. The alignment was performed with PyMol. (**C).** Surface model of the proposed EVD68 VP1 structure (blue), with clade-specific residues indicated in red. The panel on the right provides a detailed cartoon model showing clade-specific residues (in red) located on the loops forming the “canyon” responsible for host-receptor interaction. The BC-loop and DE-loop, thought to be critical in both host-receptor binding and viral antigenicity [40], are labeled with orange frames.

An interesting question arising from the novel EVD68 clade classification was why these specific 18 positions varied. To better understand the impact of these positions on the EVD68 virion, we attempted to apply these variations to previously published EVD68 structures [20]. However, many of these positions (positions 2, 92, 141, 143, 144, 145, and 148) could not be located on the model (Fig 5A). Therefore, we proposed another structural model of EVD68 VP1 based on an additional published enterovirus structure (PDB: 2X5I) [38]. Structural alignment using the PyMol software suggested that the proposed model was similar to the original EVD68 structure, despite minor differences at relaxed regions (Fig 5B). Using both structures, we were able to locate 17 positions listed in Table 2. Interestingly, 11 of these positions are located at the VP1 “canyon” formation region (Fig 5A and 5C); all 11 residues are situated on four loops, including the BC-loop and DE-loop (Fig 5C) surrounding the icosahedral five-fold axes (i.e., the “canyons”), which are considered as the most important sites for both receptor interaction [20] and neutralizing immunogenicity [40]. In addition, positions 297 and 305 are located on the surface of EVD68, and therefore, might contribute to virion antigenicity. Variations in these positions would benefit EVD68 during adaptation of local host tropism, further confirming the new classification proposed in this study.

## Discussion

Despite their size and dependence on host cells, viruses continue to evolve, through mechanisms such as ligand adaptation, immune evasion, host machinery hijacking, and/or viral enzyme improvement, in order to enhance their replication within the host. In EVD68, the former two mechanisms require variation of viral capsid proteins. The results of our present study analyzing the VP1 region of EVD68 strains from the 2014 outbreak revealed a recently derived novel EVD68 clade, Clade D, which contains unique residues at certain positions, including ones responsible for receptor and antibody recognition [40]. In addition, we propose that the TMRCA for Clade C be moved to the year 2003.6, since no strains similar to MD02-1 and MD02-2 (number 5 in Fig 1) were isolated in the US. This new clade classification provides a better interpretation of the Bayesian phylogenetic tree, since many reported EVD68 strains isolated prior to 2003 (such MD02-1 and MD02-2) stand alone instead of causing clusters or outbreaks. Sequence analysis of these unclassified strains revealed the occurrence of reverse mutations (data not shown), indicating that these strains were adapting to the host and were probably evolutionary intermediates of EVD68. Interestingly, this classification determined similar generation time points for each of the clades (2005.0, 2004.6, 2003.6, and 2005.5 for Clade A, B, C, and D, respectively), possibly indicating the existence of a yet-unknown event that occurred around 2003–2005 during the emergence of global EVD68 transmission.

Although many of the early EVD68 strains such as the Fermon reference strain were isolated in the US, no clear cluster or outbreak was detected in the US, except for one that occurred in New York City in 2009 involving Clade A strains [26]. In contrast, although all the 2014 outbreak strains clustered with strains from other countries, there was no clear evidence suggesting that they were epidemiologically linked. In particular, transmission of the Clade B strains, which were key contributors to the 2014 outbreak, most likely originated with US strain CA/AFP/11-1767 and then spread through Asia and/or Europe and back to the US where they caused the outbreak. It is also possible that CA/AFP/11-1767 continued to evolve in the US, until it was maximally adapted to the local host. Therefore, it is difficult to determine whether the strains from the 2014 outbreak were of domestic or foreign origin.

Similar to other enteroviruses, EVD68 VP1 is mostly involved in viral binding to host receptors as well as in host immune recognition [20]. Viral evolution involving VP1 is obviously persistent, since variations at four positions contributed to the severe outbreak of EVD68 in the US. These changes are most likely essential, since no previous Clade B infections were detected in the US, although many Clade B cases had been reported in Europe and Asia prior to 2014. Indeed, based on the published EVD68 structure [20], these four positions are associated with host-receptor recognition and/or antigenicity. Alterations at these positions could promote cell entry as well as enhance immune evasion. In addition to these four intra-clade variations, we identified 18 inter-clade variations in VP1, most of which were also located on the viral surface of EVD68 and were associated with “canyon” formation, supporting both the phylogenetic and serological classification of EVD68.

Our findings have important implications in terms of treatment and vaccine development for EVD68 or enteroviruses in general. In theory, preventing viral entry by blocking the “canyon”-receptor interaction would constitute the best way to cure or, at the least, control enteroviral infection. Previous studies have confirmed the role of VP1 variation as a major factor affecting enteroviral immunogenicity [9, 41]. In the case of EVD68, the anti-rhinovirus drug pleconaril has already been shown to interact with VP1 and suppress viral pathogenesis in HeLa cells [20]. The published viral inhibitors of EVA71, one of the major causative pathogens of HFMD, were also designed to interact with the “canyon” region [42]. In contrast to these previous findings, the data obtained in this study suggests that the VP1 protein is capable of changing under selective pressure; a concept applicable to EVD68 and enteroviruses in general. Moreover, the identification of the Clade B subclade indicates that such changes can occur very easily, since the four protein variations occurred within just a few years. In addition, we have previously observed that a single amino acid variation in EVA71 VP1 leads to significant differences in the host immune response [9]. Such occurrences in EVD68 (and probably other enteroviruses as well) poses challenges in the development of antivirals and/or vaccines, the success of which may require the development of novel strategies, such as the design of antiviral drugs that suppress the function of viral protease 3C [43].

In summary, we determined that Clade B EVD68 strains had the greatest impact on the 2014 outbreak in North America. Both sequence and structural analyses indicate that viral adaptation to the local host environment and/or viral evasion from host immune recognition most likely contributed to the spread of Clade B EVD68 strains in the US. In addition, a new Clade D was classified based on Bayesian evolutionary analysis, phylogenetic analysis, as well as differences in the VP1 protein. Whether this new Clade may contribute to future EVD68 transmission is an interesting question. Furthermore, detailed structural information indicated that variation in “canyon” formation in VP1 is the probable cause of the generation of different EVD68 clades and altered viral antigenicity. Multiple analyses of the VP1 protein suggest that the recently generated EVD68 strains continue to evolve and adapt to the human host. Therefore, further efforts are required to monitor EVD68 transmission and evolution in order to better control and prevent further damage caused by this emerging pathogen.

## Supporting Information

S1 Table EVD68 strains used in this study. (DOCX)
