# Supplementary material for: Analysis of Enterovirus 68 Strains from the 2014 North American Outbreak Reveals a New Clade, Indicating Viral Evolution
Source: PLoS One. 2015 Dec 2;10(12):e0144208. doi: 10.1371/journal.pone.0144208 (PMC4667938; doi:10.1371/journal.pone.0144208)
Supplement: S1 Table — (DOCX) [file pone.0144208.s001.docx]

S1 Table. EVD68 sequences used in this study.

| Strain Name | Country | Year | Accession Number |
| --- | --- | --- | --- |
| Fermon | US | 1962 | AY426531 |
| US/CA/14-6067 | US | 2014 | KP126910 |
| US/CA/14-R1 | US | 2014 | KP126909 |
| US/CA/14-R2 | US | 2014 | KP126908 |
| US/CA/14-6100 | US | 2014 | KP100796 |
| US/CA/14-6103SIB | US | 2014 | KP100795 |
| US/CO/13-60 | US | 2014 | KP100794 |
| US/CA/14-6092 | US | 2014 | KP100792 |
| US/CO/14-86 | US | 2014 | KP126912 |
| US/CO/14-94 | US | 2014 | KP100793 |
| EV-D68_STL_2014_12 | US | 2014 | KM881710 |
| CA/AFP/v14T04344 | US | 2014 | KM892502 |
| CA/AFP/11-1767 | US | 2014 | KM892501 |
| CA/RESP/10-786 | US | 2014 | KM892500 |
| CA/AFP/v12T00346 | US | 2014 | KM892499 |
| CA/AFP/v12T04950 | US | 2014 | KM892498 |
| CA/RESP/09-871 | US | 2014 | KM892497 |
| US/MO/14-18947 | US | 2014 | KM851225 |
| US/MO/14-18948 | US | 2014 | KM851226 |
| US/MO/14-18949 | US | 2014 | KM851227 |
| US/MO/14-18950 | US | 2014 | KM851228 |
| US/MO/14-18951 | US | 2014 | KM851229 |
| US/MO/14-18952 | US | 2014 | KM851230 |
| US/MO/14-18953 | US | 2014 | KM851231 |
| NZ-2010-541 | New Zealand | 2010 | JX070222 |
| NYC403 | US | 2009 | JX101846 |
| JPOC10-378 | Japan | 2010 | AB601883 |
| JPOC10-290 | Japan | 2010 | AB601882 |
| JPOC10-404 | Japan | 2010 | AB601885 |
| JPOC10-396 | Japan | 2010 | AB601884 |
| 37-99 | France | 1999 | EF107098 |
| BCH895A | China | 2010 | KF726085 |
| CA62-1 | US | 1962 | AY426486 |
| CA62-2 | US | 1962 | AY426487 |
| CA62-3 | US | 1962 | AY426488 |
| MN89 | US | 1989 | AY426489 |
| NY93 | US | 1989 | AY426490 |
| MD02-1 | US | 2002 | AY426491 |
| MD02-2 | US | 2014 | AY426492 |
| MO00 | US | 2000 | AY426493 |
| WI00 | US | 2000 | AY426494 |
| TX02-1 | US | 2002 | AY426495 |
| TX02-2 | US | 2002 | AY426496 |
| MN98 | US | 1998 | AY426497 |
| TX99 | US | 1999 | AY426498 |
| MD99 | US | 1999 | AY426499 |
| TX03 | US | 2003 | AY426500 |
| 1939-Yamagata-2010 | Japan | 2010 | AB614406 |
| 2013-Yamagata-2010 | Japan | 2010 | AB614407 |
| 1946-Yamagata-2010 | Japan | 2010 | AB614408 |
| 1975-Yamagata-2010 | Japan | 2010 | AB614409 |
| 1976-Yamagata-2010 | Japan | 2010 | AB614410 |
| 1980-Yamagata-2010 | Japan | 2010 | AB614411 |
| 1981-Yamagata-2010 | Japan | 2010 | AB614412 |
| 1989-Yamagata-2010 | Japan | 2010 | AB614413 |
| 2034-Yamagata-2010 | Japan | 2010 | AB614414 |
| 2058-Yamagata-2010 | Japan | 2010 | AB614415 |
| 2070-Yamagata-2010 | Japan | 2010 | AB614416 |
| 2071-Yamagata-2010 | Japan | 2010 | AB614417 |
| 2079-Yamagata-2010 | Japan | 2010 | AB614418 |
| 2150-Yamagata-2010 | Japan | 2010 | AB614419 |
| 2155-Yamagata-2010 | Japan | 2010 | AB614420 |
| 2158-Yamagata-2010 | Japan | 2010 | AB614421 |
| 2192-Yamagata-2010 | Japan | 2010 | AB614422 |
| 2011-Yamagata-2010 | Japan | 2010 | AB614423 |
| 2015-Yamagata-2010 | Japan | 2010 | AB614424 |
| 2016-Yamagata-2010 | Japan | 2010 | AB614425 |
| 2032-Yamagata-2010 | Japan | 2010 | AB614426 |
| 2035-Yamagata-2010 | Japan | 2010 | AB614427 |
| 2037-Yamagata-2010 | Japan | 2010 | AB614428 |
| 2052-Yamagata-2010 | Japan | 2010 | AB614429 |
| 2082-Yamagata-2010 | Japan | 2010 | AB614430 |
| 2145-Yamagata-2010 | Japan | 2010 | AB614431 |
| 2163-Yamagata-2010 | Japan | 2010 | AB614432 |
| 2086-Yamagata-2010 | Japan | 2010 | AB614433 |
| 2101-Yamagata-2010 | Japan | 2010 | AB614434 |
| 2116-Yamagata-2010 | Japan | 2010 | AB614435 |
| 2146-Yamagata-2010 | Japan | 2010 | AB614436 |
| 2161-Yamagata-2010 | Japan | 2010 | AB614437 |
| 2166-Yamagata-2010 | Japan | 2010 | AB614438 |
| 2167-Yamagata-2010 | Japan | 2010 | AB614439 |
| 2076-Yamagata-2010 | Japan | 2010 | AB614440 |
| 2093-Yamagata-2010 | Japan | 2010 | AB614441 |
| 2174-Yamagata-2010 | Japan | 2010 | AB614442 |
| 2256-Yamagata-2010 | Japan | 2010 | AB614443 |
| 2336-Yamagata-2010 | Japan | 2010 | AB614444 |
| JPOC10-200 | Japan | 2010 | AB601872 |
| JPOC10-373 | Japan | 2010 | AB601873 |
| JPOC10-402 | Japan | 2010 | AB601874 |
| JPOC10-412 | Japan | 2010 | AB601875 |
| JPOC10-441 | Japan | 2010 | AB601876 |
| JPOC10-445 | Japan | 2010 | AB601877 |
| JPOC10-471 | Japan | 2010 | AB601878 |
| JPOC10-573 | Japan | 2010 | AB601879 |
| JPOC10-616 | Japan | 2010 | AB601880 |
| JPOC10-618 | Japan | 2010 | AB601881 |
| EV68_NL_201013226 | Netherlands | 2010 | JF896287 |
| EV68_NL_201014542 | Netherlands | 2010 | JF896288 |
| EV68_NL_201013352 | Netherlands | 2010 | JF896289 |
| EV68_NL_201012910 | Netherlands | 2010 | JF896290 |
| EV68_NL_201012462 | Netherlands | 2010 | JF896291 |
| EV68_NL_201014502 | Netherlands | 2010 | JF896292 |
| EV68_NL_201012159 | Netherlands | 2010 | JF896293 |
| EV68_NL_201012721 | Netherlands | 2010 | JF896294 |
| EV68_NL_201012756 | Netherlands | 2010 | JF896295 |
| EV68_NL_201012463 | Netherlands | 2010 | JF896296 |
| EV68_NL_201011288 | Netherlands | 2010 | JF896297 |
| EV68_NL_201012233 | Netherlands | 2010 | JF896298 |
| EV68_NL_201012584 | Netherlands | 2010 | JF896299 |
| EV68_NL_201012472 | Netherlands | 2010 | JF896300 |
| EV68_NL_201012493 | Netherlands | 2010 | JF896301 |
| EV68_NL_201013557 | Netherlands | 2010 | JF896302 |
| EV68_NL_201012467 | Netherlands | 2010 | JF896303 |
| EV68_NL_201011595 | Netherlands | 2010 | JF896304 |
| EV68_NL_201014073 | Netherlands | 2010 | JF896305 |
| EV68_NL_201013230 | Netherlands | 2010 | JF896306 |
| EV68_NL_201012867 | Netherlands | 2010 | JF896307 |
| EV68_NL_200913563 | Netherlands | 2009 | JF896308 |
| EV68_NL_200914986 | Netherlands | 2009 | JF896309 |
| EV68_NL_200914918 | Netherlands | 2009 | JF896310 |
| EV68_NL_200912598 | Netherlands | 2009 | JF896311 |
| EV68_NL_201013421 | Netherlands | 2010 | JF896312 |
| 2124-Yamagata-2005 | Japan | 2005 | AB667892 |
| 2062-Yamagata-2005 | Japan | 2005 | AB667891 |
| 2050-Yamagata-2005 | Japan | 2005 | AB667890 |
| 2043-Yamagata-2005 | Japan | 2005 | AB667889 |
| 2038-Yamagata-2005 | Japan | 2005 | AB667888 |
| 2037-Yamagata-2005 | Japan | 2005 | AB667887 |
| 1991-Yamagata-2005 | Japan | 2005 | AB667886 |
| 1989-Yamagata-2005 | Japan | 2005 | AB667885 |
| 1737-Yamagata-2008 | Japan | 2008 | AB667899 |
| 1833-Yamagata-2008 | Japan | 2008 | AB667898 |
| 2118-Yamagata-2007 | Japan | 2007 | AB667897 |
| 2311-Yamagata-2006 | Japan | 2006 | AB667896 |
| 1703-Yamagata-2007 | Japan | 2007 | AB667895 |
| 2251-Yamagata-2005 | Japan | 2005 | AB667894 |
| 2218-Yamagata-2005 | Japan | 2005 | AB667893 |
| ARI192 | US | 2009 | JX101786 |
| SEN03 | Senegal | 2010 | JX101787 |
| SEN30 | Senegal | 2010 | JX101788 |
| SEN37 | Senegal | 2010 | JX101789 |
| GA420 | Gambia | 2008 | JX101790 |
| GA421 | Gambia | 2008 | JX101791 |
| GA424 | Gambia | 2008 | JX101792 |
| GA427 | Gambia | 2008 | JX101793 |
| GA431 | Gambia | 2008 | JX101794 |
| SA402 | South Africa | 2001 | JX101795 |
| SA498 | South Africa | 2001 | JX101796 |
| SA551 | South Africa | 2000 | JX101797 |
| SA553 | South Africa | 2000 | JX101798 |
| SA563 | South Africa | 2000 | JX101799 |
| SA792 | South Africa | 2000 | JX101800 |
| SA726 | South Africa | 2000 | JX101801 |
| SA1354 | South Africa | 2000 | JX101802 |
| NYC369 | US | 2009 | JX101803 |
| NYC394 | US | 2009 | JX101804 |
| NYC399 | US | 2009 | JX101805 |
| NYC442 | US | 2009 | JX101807 |
| NYC409 | US | 2009 | JX101808 |
| NYC435 | US | 2009 | JX101809 |
| NYC458 | US | 2009 | JX101810 |
| NYC465 | US | 2009 | JX101811 |
| NYC496 | US | 2009 | JX101812 |
| NYC567 | US | 2009 | JX101813 |
| NYC817 | US | 2009 | JX101814 |
| NZ-2010-1507 | New Zealand | 2010 | JQ713904 |
| NZ-2010-183 | New Zealand | 2010 | JQ713905 |
| NZ-2010-358 | New Zealand | 2010 | JQ713906 |
| NZ-2010-404 | New Zealand | 2010 | JQ713907 |
| NZ-2010-431 | New Zealand | 2010 | JQ713908 |
| NZ-2010-435 | New Zealand | 2010 | JQ713909 |
| NZ-2010-481 | New Zealand | 2010 | JQ713910 |
| NZ-2010-539 | New Zealand | 2010 | JQ713911 |
| NZ-2010-571 | New Zealand | 2010 | JQ713913 |
| ITA/18641/08 | Italy | 2008 | KC763157 |
| ITA/19179/08 | Italy | 2008 | KC763158 |
| ITA/19391/12 | Italy | 2012 | KC763159 |
| ITA/19962/08 | Italy | 2008 | KC763160 |
| ITA/20260/08 | Italy | 2008 | KC763161 |
| ITA/20528/12 | Italy | 2012 | KC763162 |
| ITA/22289/08 | Italy | 2008 | KC763163 |
| ITA/22516/12 | Italy | 2012 | KC763164 |
| ITA/22719/08 | Italy | 2008 | KC763165 |
| ITA/23352/08 | Italy | 2008 | KC763166 |
| ITA/23695/12 | Italy | 2012 | KC763167 |
| ITA/24281/08 | Italy | 2008 | KC763168 |
| ITA/24518/12 | Italy | 2012 | KC763169 |
| ITA/26423/08 | Italy | 2008 | KC763170 |
| ITA/26505/08 | Italy | 2008 | KC763171 |
| ITA/26868/08 | Italy | 2008 | KC763172 |
| ITA/27708/08 | Italy | 2008 | KC763173 |
| ITA/31742/10 | Italy | 2010 | KC763174 |
| ITA/33658/10 | Italy | 2010 | KC763175 |
| ITA/33707/10 | Italy | 2010 | KC763176 |
| ITA/34800/10 | Italy | 2010 | KC763177 |
| CQ4278 | China | 2010 | JX898784 |
| CQ5585 | China | 2011 | JX898785 |
| CQ5914 | China | 2012 | JX898786 |
| ID72 | Spain | 2013 | KF254913 |
| SO9770 | Spain | 2013 | KF254914 |
| SO9749 | Spain | 2013 | KF254915 |
| SO9336 | Spain | 2012 | KF254917 |
| SO9493 | Spain | 2012 | KF254918 |
| SO9320 | Spain | 2012 | KF254919 |
| SO9277 | Spain | 2012 | KF254920 |
| SO9411 | Spain | 2012 | KF254921 |
| SO9288 | Spain | 2012 | KF254922 |
| SO9306 | Spain | 2012 | KF254923 |
| SO9406 | Spain | 2012 | KF254924 |
| HEV044008 | Kenya | 2008 | KJ472878 |
| HEV085008 | Kenya | 2008 | KJ472880 |
| HEV156010 | Kenya | 2010 | KJ472882 |
| HEV124010 | Kenya | 2010 | KJ472883 |
| HEV126010 | Kenya | 2010 | KJ472884 |
| HEV137010 | Kenya | 2010 | KJ472885 |
| HEV196011 | Kenya | 2011 | KJ472886 |
